# Author Correction: RPAP3 provides a flexible scaffold for coupling HSP90 to the human R2TP co-chaperone complex

**DOI:** 10.1038/s41467-018-05546-1

**Published:** 2018-07-31

**Authors:** Fabrizio Martino, Mohinder Pal, Hugo Muñoz-Hernández, Carlos F. Rodríguez, Rafael Núñez-Ramírez, David Gil-Carton, Gianluca Degliesposti, J. Mark Skehel, S. Mark Roe, Chrisostomos Prodromou, Laurence H. Pearl, Oscar Llorca

**Affiliations:** 1 Centro de Investigaciones Biológicas (CIB), Spanish National Research Council (CSIC), Ramiro de Maeztu 9, 28040 Madrid, Spain; 2 Genome Damage and Stability Centre, School of Life Sciences, University of Sussex, Falmer, BN1 9RQ Brighton, UK; 3 Spanish National Cancer Research Centre (CNIO), Melchor Fernández Almagro 3, 28029 Madrid, Spain; 4 Structural Biology Unit, CIC bioGUNE, Bizkaia Technology Park, 48160 Derio, Spain; 5 MRC Laboratory of Molecular Biology, Cambridge Biomedical Campus, Francis Crick Avenue, CB2 0QH Cambridge, UK

Correction to: *Nature Communications* 10.1038/s41467-018-03942-1; published online 16 April 2018

In the originally published version of this article, the affiliation details for Hugo Muñoz-Hernández, Carlos F. Rodríguez and Oscar Llorca incorrectly omitted ‘Centro de Investigaciones Biológicas (CIB), Spanish National Research Council (CSIC), Ramiro de Maeztu 9, 28040 Madrid, Spain’. This has now been corrected in both the PDF and HTML versions of the Article.

