# Supplementary material for: RPAP3 provides a flexible scaffold for coupling HSP90 to the human R2TP co-chaperone complex
Source: Nat Commun. 2018 Apr 16;9:1501. doi: 10.1038/s41467-018-03942-1 (PMC5902453; doi:10.1038/s41467-018-03942-1)
Supplement: Supplementary file 1 — Suplementary Information [file 41467_2018_3942_MOESM1_ESM.docx]

**SUPPLEMENTARY INFORMATION**

**RPAP3 provides a flexible scaffold for coupling HSP90 to the human R2TP co-chaperone complex**

‘Martino et al.’

**
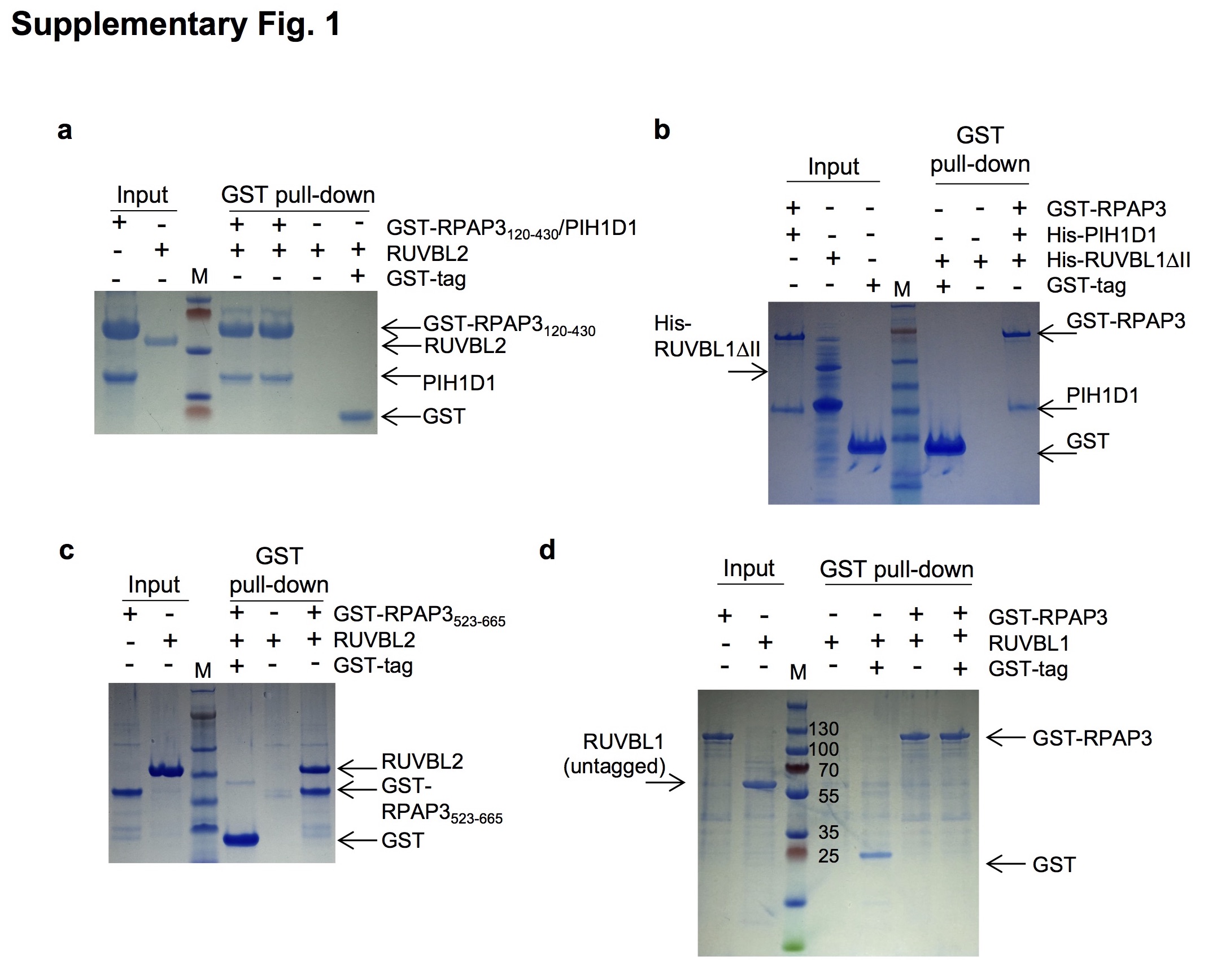
**

**Supplementary Fig. 1.** Interactions between R2TP components. Complementary experiments to those shown in Fig. 1. The figure shows control experiments for the GST tag, and the results when the tags in RUVBL1 were removed.

(a) Pull down experiments showing that PIH1D1 can be co-purified with a fragment of RPAP3 comprising residues 120-430, but this complex does not bind RUVBL2. “M” indicates molecular weight markers.

(b) Pull down experiments showing that full length RPAP3 co-purified with PIH1D1 do not interact with RuvBL1 with the DII domain truncated. Similar results were found for full length RuvBL1 (**Fig. 1d**). “M” indicates molecular weight markers.

(c) Pull down experiments depicting the interactions between the RBD domain in RPAP3 and RUVBL2. M indicates molecular weight markers hereafter.

(d) Similar pull-down experiments were performed using full length untagged RUVBL1. These experiments showed that the tag in RUVBL1 has no effect on the absence of interaction with RPAP3, since both tagged and untagged RUVBL1 behaved similarly.


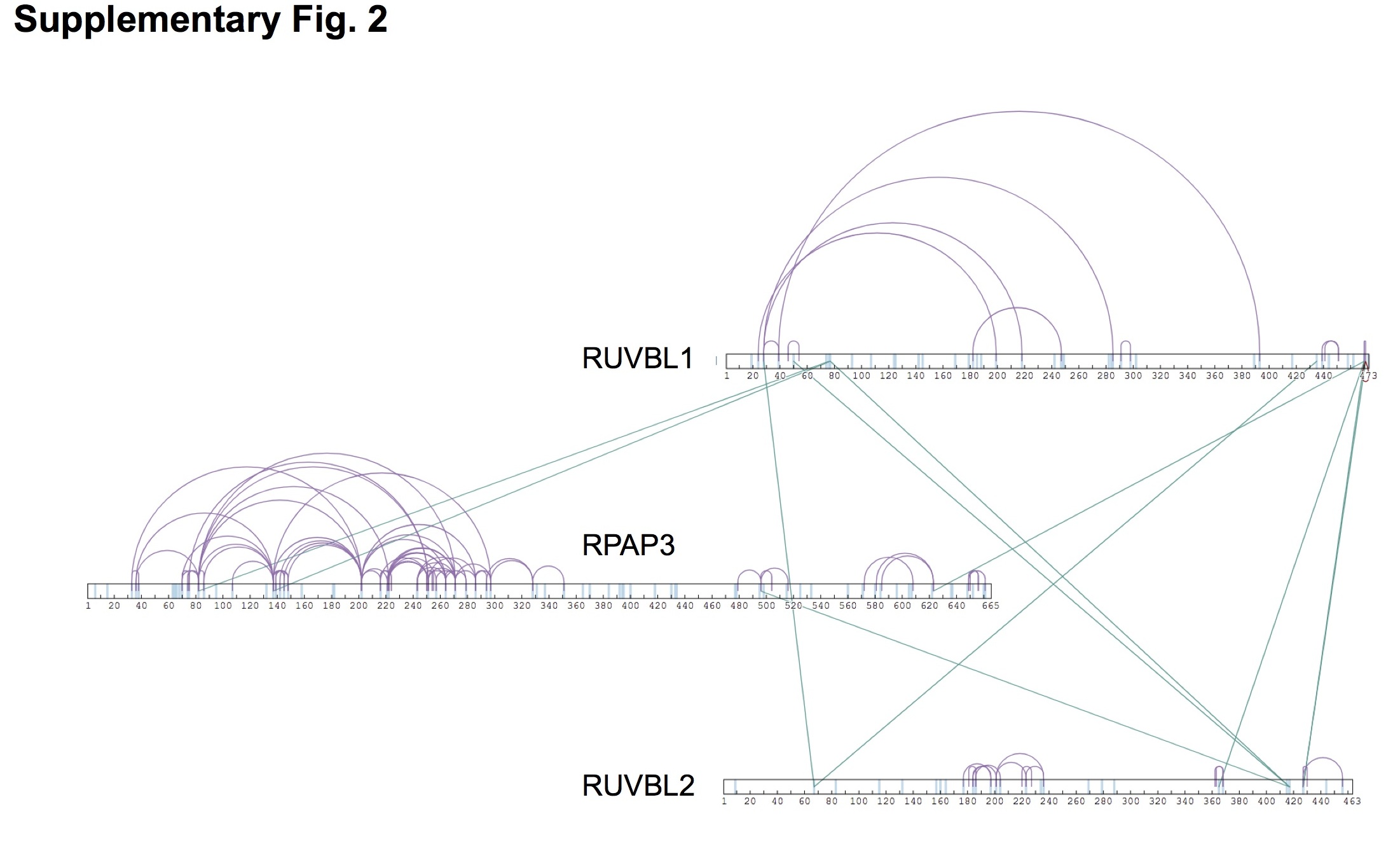


**Supplementary Fig. 2.** Cross-linking mass spectrometry (XL-MS) for human R2TP complex. The experiments performed detected crosslinks between RUVBL1 and RUVBL2 that were consistent with the crystal structure of the RUVBL1-RUVBL2 hetero-hexamers, serving as internal validation of the results obtained. Crosslinks between RUVBL1 and RPAP3, and RUVBL2 and RPAP3 are shown in detail in Fig. 4b. XL-MS did not detect crosslinks between PIH1D1 and any other component in the complex.


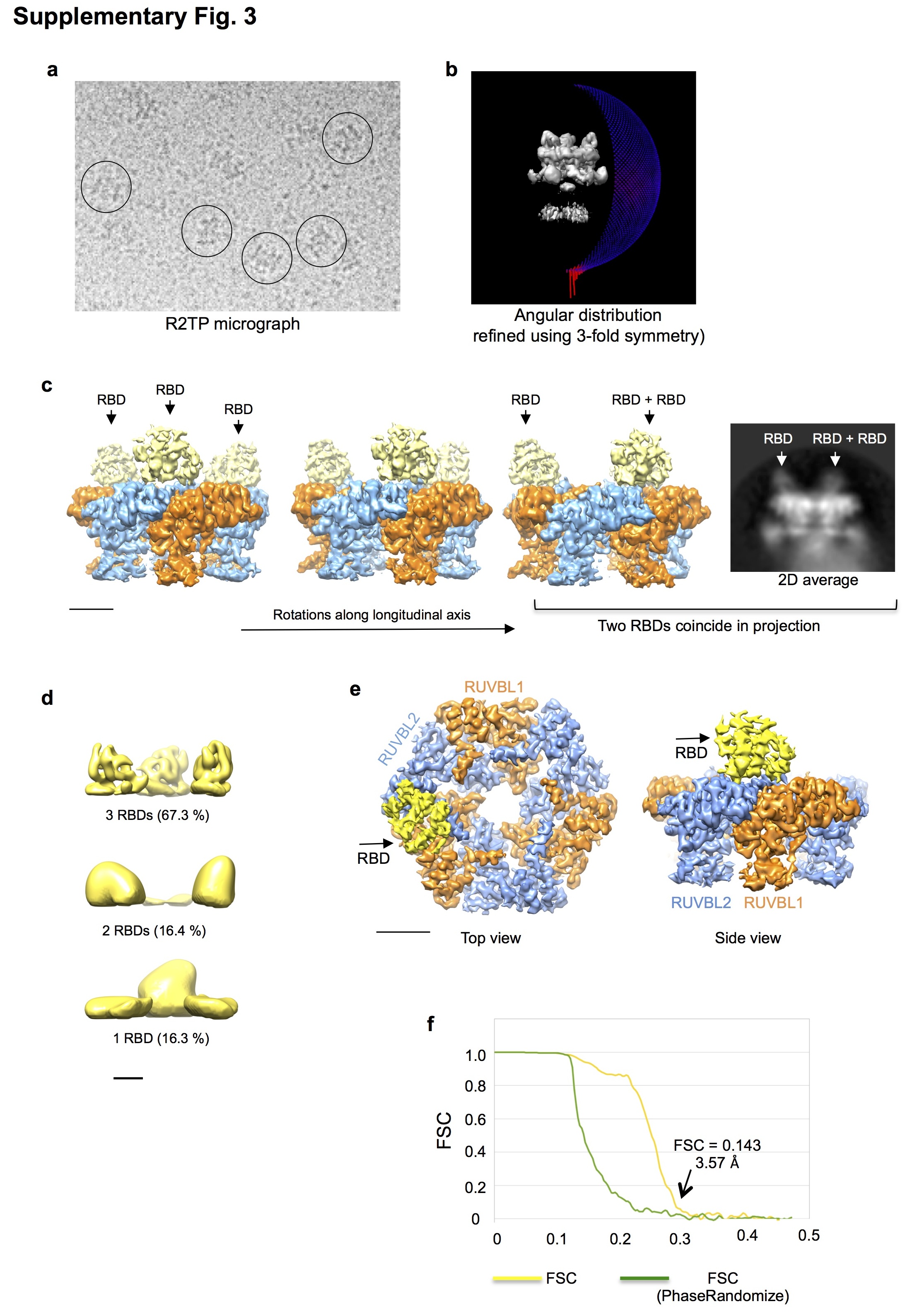


**Supplementary Fig. 3.** Cryo-EM and high resolution processing of RUVBL1-RUVBL2-RBD

(a) Representative micrograph of R2TP observed using cryo-EM, grids without a carbon support film, a FEI Titan Krios microscope (eBIC, Oxford, UK, EU) and a K2 detect.

(b) Angular distribution of views of R2TP in cryo-EM covers the whole sphere. The structure shown was processed using 3-fold rotational symmetry, and therefore, only part of the sphere is shown.

(c) 2D average and one view of RUVBL1-RUVBL2-RBD cryo-EM map containing 3 RBDs, showing that some orientations of the map generate projections where only 2 RBD domains are apparent, because 2 RBDs coincide in the projection. Scale bar, 2.5 nm.

(d) After extensive 3D classification of the R2TP images, and using a mask around the RBD region, particles were grouped in three classes, containing 1, 2 or 3 RBDs, being the latest group the most abundant and better quality. A representative volume for each class is shown. Scale bar, 2.5 nm.

(e) Two views of the RUVBL1-RUVBL2-RBD structure obtained following the protocols described in Methods. The structure of 1 RBD bound to the RUVBL ring was determined using these procedures. Scale bar, 2.5 nm.

(f) Resolution estimation of the structure of RUVBL1-RUVBL2-RBD, using FSC and the gold standard as defined in RELION.


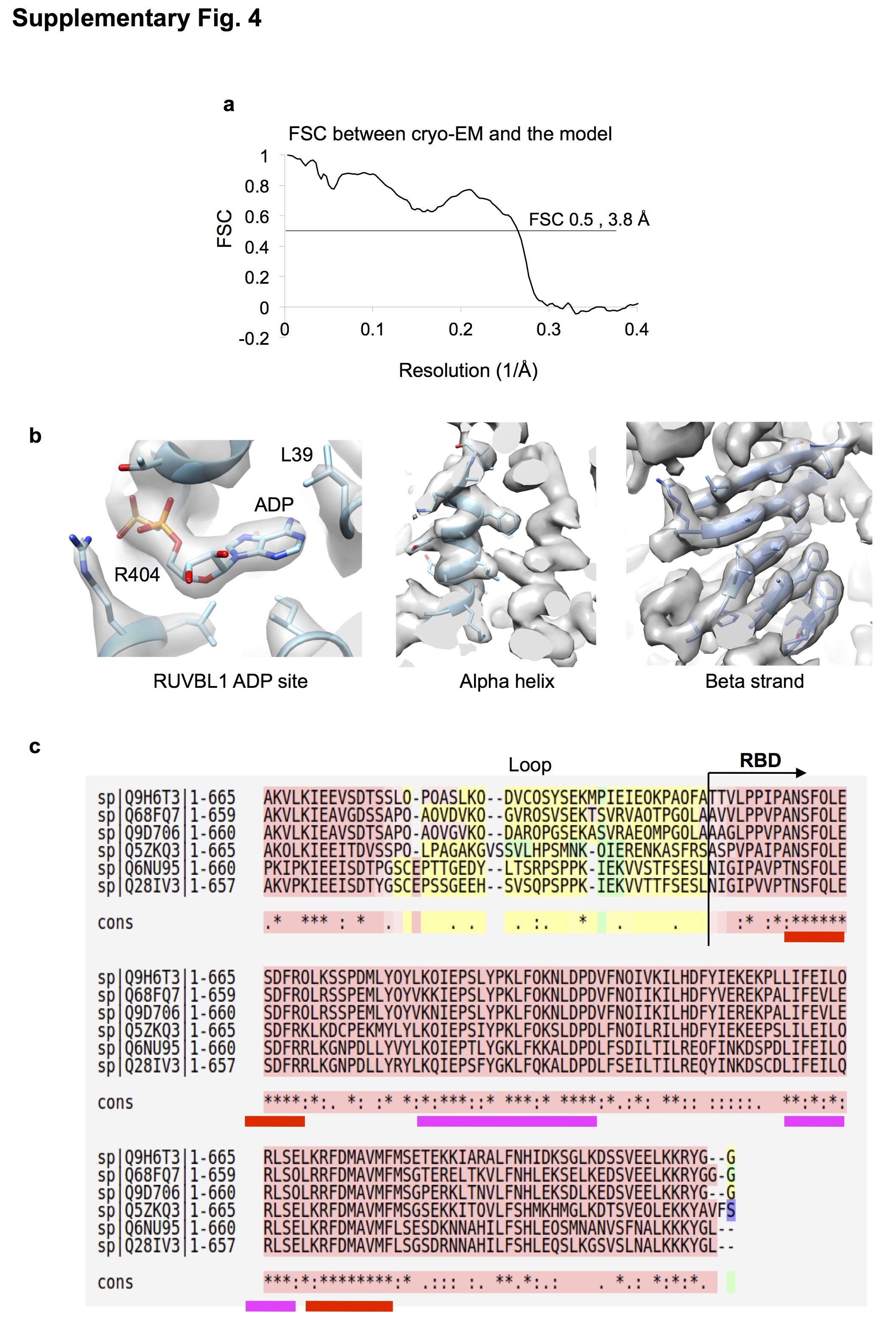


**Supplementary Fig. 4.** High-resolution details of the RUVBL1-RUVBL2-RBD complex.

(a) FSC curve comparing the atomic model and the cryo-EM density.

(b) Selection of elements of secondary structure and side chains that are well resolved in the structure of RUVBL1-RUVBL2 within R2TP. The ADP in the map is also clearly resolved.

(c) Comparison of the sequence of the RBD from several species (Human, Rat, Mice, Chicken and Xenopus), showing that H1 and H6 are 100 % identical (**Figure 6b**, red colour). Other well-conserved regions are labelled in pink colour.


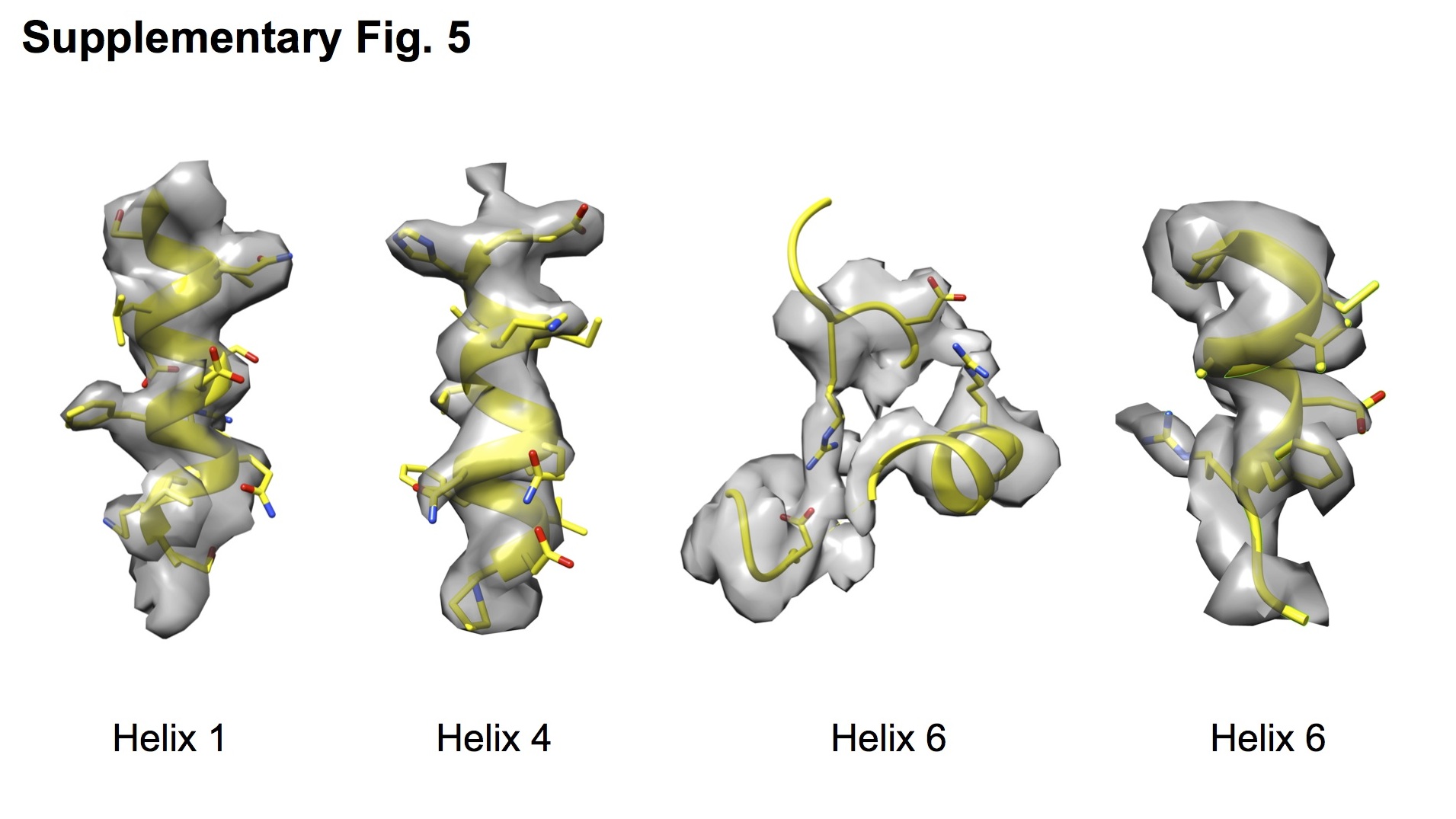


**Supplementary Fig. 5.** High-resolution details of the RBD domain, showing density for side chains.

**
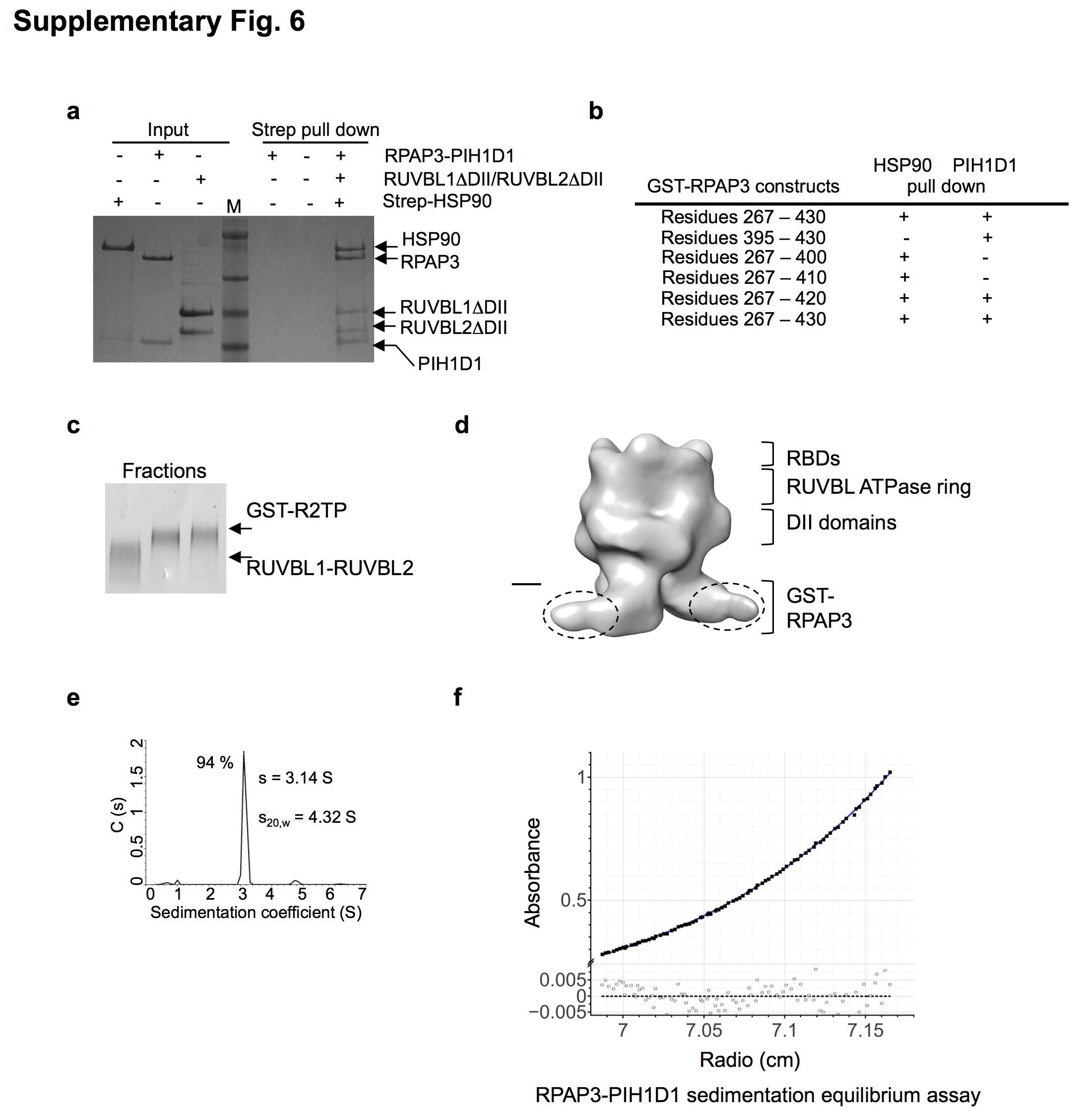
**

**Supplementary Fig. 6.** Analysis of the flexible regions in R2TP

(a) Pull down experiments using Strep-HSP90 showing that HSP90 can interact with the R2TP reconstituted in vitro from the RUVBL1-RUVBL2 and RPAP3-PIH1D1 sub-complexes. The DII domains can be deleted without affecting the assembly of R2TP or the interaction with HSP90.

(b) Several GST-RPAP3 constructs were tested for the capacity to bind PIH1D1 that can be expressed and purified soluble. Interactions were analysed by GST pull down of the RPAP3 construct, and the presence of HSP90 or PIH1D1 analysed. Results are summarized in the table.

(c) Stabilization and purification of R2TP using GA cross-linking and sucrose gradients, and GST-RPAP3 (GST tag present in N-terminus). RUVBL1-RUVBL2 and GST-RPAP3-PIH1D1 complexes were mixed, using an excess of GST-RPAP3-PIH1D1, and the mixture was resolved in a sucrose gradient. RPAP3-PIH1D1 runs in slower migration fractions of the gradient, compared to R2TP. Fractions from the gradient were analysed using BLUE-NATIVE electrophoresis, and the GST-labelled R2TP appeared as a defined band. Cross-linked R2TP was analysed at low resolution using negative stain and cryo-EM.

(d) GST-labelling of the N-terminus of RPAP3 in R2TP. Cryo-EM images of the selected fraction of GST-R2TP were collected and processed applying 3-fold symmetry. The resulting low-resolution structure was sufficient to identify a ring reminiscent of RUVBL1-RUVBL2 decorated by RBDs, whereas the opposite end showed protrusions compatible with the GST. Scale bar, 2.5 nm.

(e) In sedimentation velocity experiments 94 % of the TP sample was observed as a species with an experimental sedimentation coefficient of 3.1S. This value corrected to standard conditions (s_20,w_ = 4.3S) was compatible with an elongated (f/f_0_ = 1.8) heterodimer (1RPAP3:1PIH1D1).

(f) To confirm this result, sedimentation equilibrium assays were carried out under the same experimental conditions. The calculated average molecular mass obtained was 103,900 ± 320 Da, which corresponds to the abovementioned heterodimer (104,212 Da).


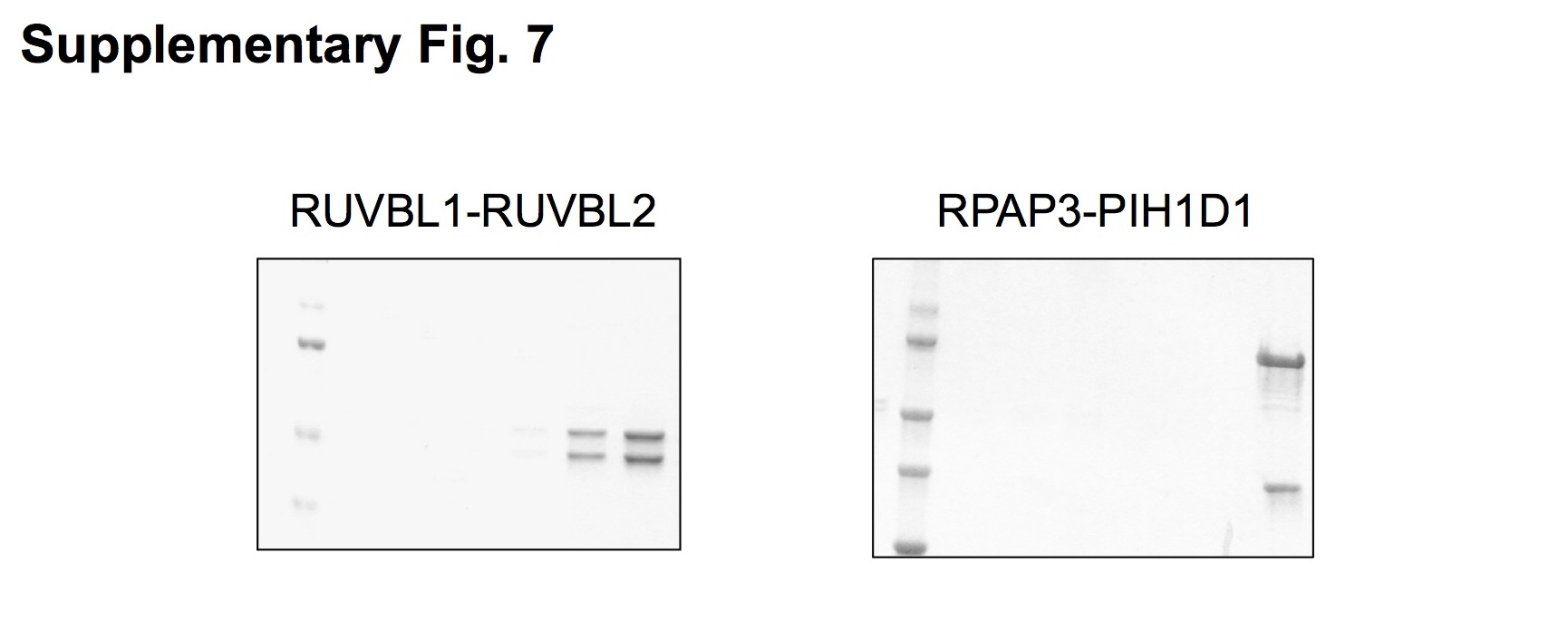


**Supplementary Fig. 7.** Complete SDS-PAGE gels for panels shown in Fig. 3b. Each lane correspond to a fraction of a size-exclusion chromatography in the final step of the purification of each of these two complexes.

**Supplementary Table 1.**

|  | **Primer 5’-3’** |
| --- | --- |
| Hsp90 FL alpha Forward | TCCAGGGGCCCCATATGATGCCTGAGGAAACCCAGACCCAAGACCAA |
| Hsp90 FL alpha Reverse | GTCATGCTAGCCATATGTTAGTCTACTTCTTCCATGCGTGATGTGTCGTC |
| Hsp90 FL beta Forward | TCCAGGGGCCCCATATGATGCCTGAGGAAGTGCACCATGGAGAGGAG |
| Hsp90 FL beta Reverse | GTCATGCTAGCCATATGTTAATCGACTTCTTCCATGCGAGACGCATCCTC |
| RPAP3 400 Forward | CCCTGGGATCTCATATGGGTCATTGGGATGACGTGTTCCTG |
| RPAP3 420 Forward | CCCTGGGATCTCATATGGACAATCCGCCGCACCCGGGCTCC |
| RPAP3 665 Reverse | GTCATGCTAGCCATATGTAAACCGCCGTAGCGTTTTTTCAGTTCTTCGAC |
| hPIH 180C Forward | CCCTGGGATCTCATATGTCGGAGCAGCGTCCTCGGATCCAGGAGCTG |
| hPIH 180C Reverse | GTCATGCTAGCCATATGTTAAGAAGGCACCGGCAGAAGCGGCATGGCCAC |
| RPAP3 410 Forward | GATAGTACCCAGCGCCAAAACGTGGTT |
| RPAP3 665 Reverse | TAAACCGCCGTAGCGTTTTTTCAGTTCTTCGAC |
| RPAP3 430 Stop Forward | CCGGGCTCCACGTAACCGCTGAAGAAAGTGATT |
| RPAP3 430 Stop Reverse | AATCACTTTCTTCAGCGGTTACGTGGAGCCCGG |
| RPAP3 Forward | CCCTGGGATCTCATATGACCTCGGCAAACAAAGCCATCGAACTGCAA |
| RPAP3 121 Forward | CCCTGGGATCTCATATGAGCGAATCTGAAGAAGATGGCATTCACGTG |
| RPAP3 267 Forward | CCCTGGGATCTCATATGGAAGGCGAACGTAAACAGATTGAAGCTCAG |
| RPAP3 430 Reverse | CGTTACTAGTGGATCCTTATTTCGTGGAGCCCGGGTGCGGCGGATTGTCAAT |
| RPAP3 395 Forward | CCCTGGGATCTCATATGAGTAAAATTAAGAAAGAACTGATCGAA |
| RPAP3 665 Reverse | CGTTACTAGTGGATCCTTAACCGCCGTAGCGTTTTTTCAGTTCTTCGACGGAACT |
| RPAP3 430 Forward | AGGAGATATACCATGAAGAAAGTGATTATCGAAGAAACCGGT |
| RPAP3 665 Reverse | CAGAACTTCCAGTTTACCGCCGTAGCGTTTTTTCA |
| RPAP3 545 Forward | AGGAGATATACCATGGCGAACAGTTTTCAGCTGGAAT |
| RPAP3 541 Forward | AGGAGATATACCATGACCACGGTGCTGCCG |

Primer sequences used in this study

**Supplementary Table 2.**

| **Cryo-EM of R2TP-1RBD and modelling** | (EMDB-4287)  (PDB 6FO1) |  |
| --- | --- | --- |
| **Data collection and processing** |  |  |
| Microscope | FEI Titan Krios |  |
| Detector | Gatan K2 (counting mode) |  |
| Calibrated magnification | 47170 |  |
| Voltage (kV) | 300 |  |
| Electron exposure (e–/Å^2^) | 52 |  |
| Defocus range (μm) | -1.2 to -3.0 |  |
| Pixel size (Å) | 1.06 |  |
| Symmetry imposed | C1 |  |
| Initial particle images (no.) | 96406 |  |
| Final particle images (no.) | 101742 (after C3 rotation and classification) |  |
| FSC threshold | 0.143 |  |
| Map resolution (Å) | 3.57 |  |
| Map resolution range (Å) | 3.0-5.0 |  |
|  |  |  |
| **Refinement** |  |  |
| Refinement software | phenix.real_space_refine |  |
| Initial model used (PDB code) | 2XSZ plus I-Tasser model fitted with Amber |  |
| Map sharpening *B* factor (Å^2^) | -112.011 |  |
| Model composition  Non-hydrogen atoms  Protein residues  Ligands | 15647  2016  6 ADP |  |
| *B* factors (Å^2^)  Average | 78.59 |  |
| R.m.s. deviations  Bond lengths (Å)  Bond angles (°) | 0.0007  0.724 |  |
| Validation  MolProbity score  Clashscore  Poor rotamers (%)  Favorable rotamers (%) | 1.97  11.11  0.00  97.06 |  |
| Ramachandran plot  Favored (%)  Allowed (%)  Disallowed (%) | 93.82  6.13  0.05 |  |

Mask CC 0.831

Cryo-EM data collection, refinement and validation statistics for RUVBL1-RUVBL2-RBD model, corresponding to cryo-EM map named R2TP-1RBD in text and EMDB.

**Supplementary Table 3.**

| **Processing of R2TP-subgroup1 (EMD-4290)** | |
| --- | --- |
| Software | Relion 2.1-beta-1 |
| Number of particles in final reconstruction: | 27385 |
| Map sharpening B factor (Å^2^) | -500.0 |
| Final resolution | 8.72 |

| **Processing of R2TP-subgroup2 (EMD-4291)** | |
| --- | --- |
| Software | Relion 2.1-beta-1 |
| Number of particles in final reconstruction: | 182351 |
| Map sharpening B factor (Å^2^) | -173.0 |
| Final resolution | 6.57 |

Summary of image processing of flexible regions, shown in Figure 7. R2TP-subgroup1 is shown in Fig. 7a and R2TP-subgroup2 is shown in Fig. 7b. Cryo-EM data collection details for these structures are the same as in Table 1 since these are structures obtained after classification of the same initial data set.

**Supplementary Table 4**

**Data collection and refinement statistics**

|  | RPAP3 C-terminal motif |  |
| --- | --- | --- |
| **Data collection** |  |  |
| Space group | P 4_3_ 2_1_ 2 |  |
| Cell dimensions |  |  |
| *a*, *b*, *c* (Å) | 39.631, 39.631, 70.426 |  |
| α, β, γ (°) | 90, 90, 90 |  |
| Resolution (Å) | 70.43-1.78 (1.83-1.78) |  |
| *R*_merge_ | 0.113 (0.872) |  |
| *I* / σ*I* | 8.9 (2.1) |  |
| Completeness (%) | 99.9 (100.0) |  |
| Redundancy | 8.7 (9.0) |  |
|  |  |  |
| **Refinement** |  |  |
| Resolution (Å) | 34.54-1.78 |  |
| No. reflections | 5844 |  |
| *R*_work_ / *R*_free_ | 23.3 / 27.0 |  |
| No. atoms |  |  |
| Protein | 393 |  |
| Ligand/ion | 0 |  |
| Water | 41 |  |
| *B*-factors |  |  |
| Protein | 34.9 |  |
| Ligand/ion | 0 |  |
| Water | 52.6 |  |
| R.m.s. deviations |  |  |
| Bond lengths (Å) | 0.010 |  |
| Bond angles (°) | 1.09 |  |

Values in parentheses are for highest-resolution shell.
