# Supplementary material for: RPAP3 provides a flexible scaffold for coupling HSP90 to the human R2TP co-chaperone complex
Source: Nat Commun. 2018 Apr 16;9:1501. doi: 10.1038/s41467-018-03942-1 (PMC5902453; doi:10.1038/s41467-018-03942-1)
Supplement: Supplementary file 3 — Description of Additional Supplementary Information [file 41467_2018_3942_MOESM3_ESM.docx]

**Description of Additional Supplementary Files**

File Name: Supplementary Movie 1

Description:

Movie describing the fitting of the I-TASSER prediction (red color) for the RBD domain into the cryo-EM map (transparency), as described in the Methods section. Initial frames of the movie show the result of fitting the prediction as a rigid body into the cryoEM density for the RBD domain, and showing a good agreement between the disposition of secondary structure elements in the prediction and the map. The fitting is then improved using molecular dynamics (MD) simulations in AMBER (http://ambermd.org). The last snapshot of (the 5 ns) MD simulation was then used as the starting point for modelling of RBD and RUVBL1-RUVBL2-RBD
